# Cartilaginous Fibrous Osteoma of the Ethmoid Sinus

**DOI:** 10.7759/cureus.19378

**Published:** 2021-11-08

**Authors:** Wesam Alkhatatba, Saad Alqarni

**Affiliations:** 1 Otorhinolaryngology – Head and Neck Surgery, Dr. Soliman Fakeeh Hospital, Jeddah, SAU; 2 Otorhinolaryngology – Head and Neck Surgery, King Fahad Armed Forces Hospital, Jeddah, SAU

**Keywords:** fibrous osteoma, ethmoid sinus, paranasal, cartilaginous fibrous osteoma, fibro-osseous, fibrous dysplasia

## Abstract

Fibro-osseous lesions are common in the paranasal sinuses. The incidence of fibrous dysplasia (FD) in the ethmoid sinus is rare. Patients with such lesions are usually asymptomatic until the lesion is large enough to start compressing adjacent structures and organs. Common presentations include nasal obstruction, headache, eye swelling, and diplopia. Meanwhile, less common signs can include decreased visual acuity. We present a case of a 65-year-old male with comorbidities who presented to the clinic complaining of a chronic nasal obstruction, headache, and decreased visual acuity in the right eye. On endoscopic examination, a lesion was identified in the ethmoid sinus. Computed tomography was performed and confirmed the positioning of the lesion within the ethmoid sinus compressing the optic nerve. Total excision was performed through a direct nasal endoscopic approach. The lesion was excised completely with no recurrence. Histopathology report confirmed the lesion to be of cartilaginous nature, and a final diagnosis of cartilaginous fibrous osteoma was made. Such lesions are usually benign and symptomless. Excising the lesion completely is the best approach to decrease the chances of recurrence.

## Introduction

The ethmoid sinus is one of the four paired paranasal sinuses in the human body. It is formed from air cells within the ethmoid bone between the eyes and nose [1]. Paranasal fibro-osseous lesions are benign, common, slow-growing bony and fibrous growths that occur mainly in the frontal and ethmoid sinuses and are often asymptomatic [2]. They are divided into fibrous dysplasia (FD), osteomas, and ossifying fibromas [3]. FD tend to occur in young adolescents, with an equal male/female ratio [4]. FD are found to be polyostotic in 70% of patients and monostotic in the remaining 30% with a 0.5% chance of malignant transformation, which is often among the polyostotic group [4]. Symptomatic patients often present with a headache, facial pain at the site of the lesion, facial deformities, and visual complications. Once tumors expand and start displacing the orbital cavity, they give rise to ocular symptoms such as diplopia, exophthalmos, and proptosis [5]. Nonetheless, they rarely present with adverse complications such as meningitis, intracranial mucoceles, and brain abscesses [6].

Radiologically, X-rays cannot provide sufficient details of the tumor. Meanwhile, magnetic resonance imaging (MRI) can give inaccurate results due to bone and tissue delineation [6]. Computed tomography (CT) is the golden standard imaging modality of choice as it provides an accurate estimation of the size, location, and concurrent pathology of such lesions [7]. FD are often found in the CT scan as radiolucent and sclerotic zones depending on the intensity of the fibrous and osseous tissues with a ground glass appearance [4]. Histopathologically, FD are a replacement of the normal bone by fibrous tissue [4]. Surgery is often recommended for patients who are symptomatic with rapidly growing tumors to eliminate any threat to vital adjacent organs [6]. An endoscopic sinus excision is usually performed to excise lesions isolated to the ethmoid sinus with recent evidence recommending the transnasal endoscopic approach [4]. The final diagnosis of fibro-osseous lesions is done collectively by presentation, radiology, and the final histopathology as they can share the same characteristics [3,4]. We report a case of an isolated ethmoidal sinus lesion that was diagnosed histopathologically as FD.

## Case presentation

**Figure 1:**
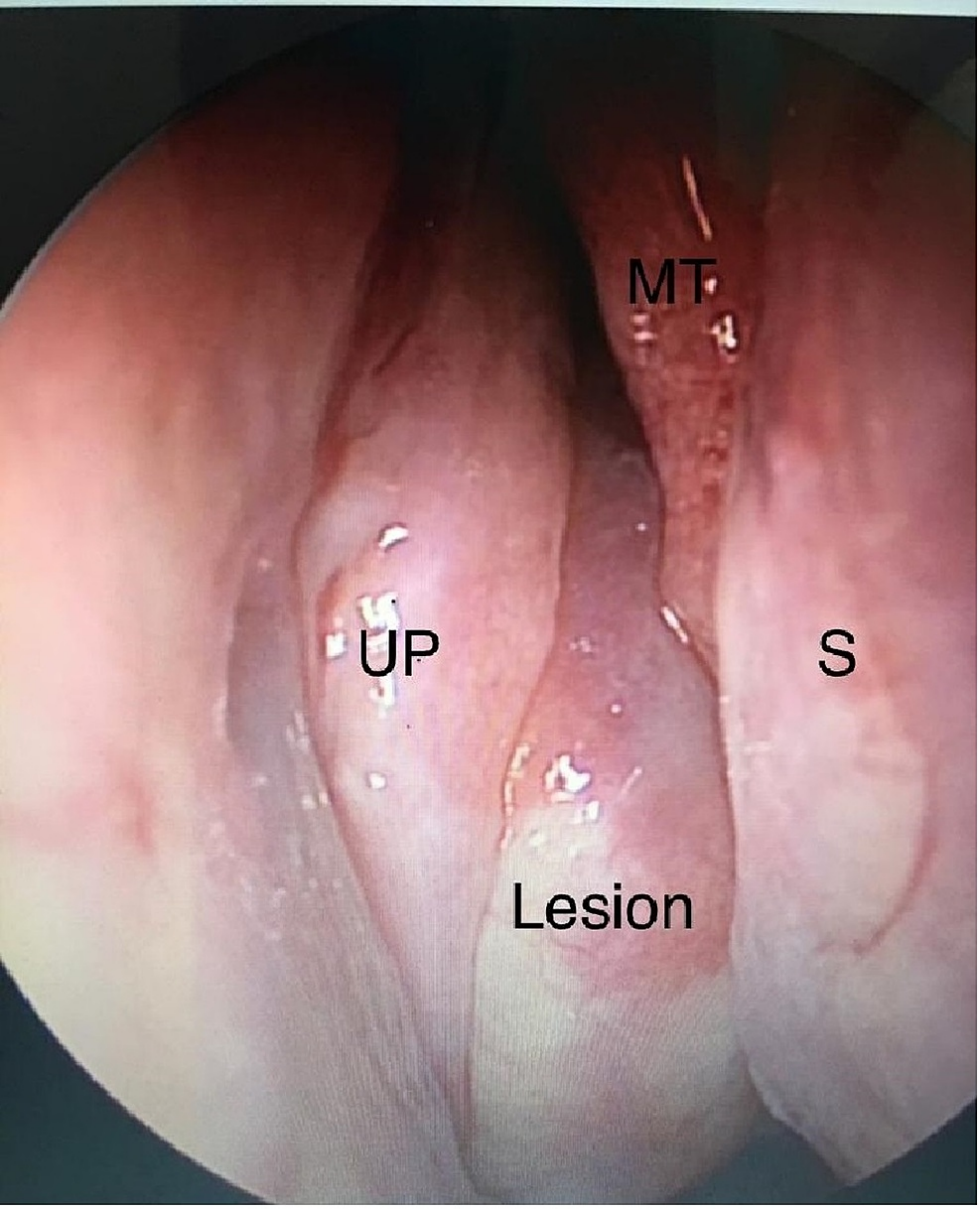
Endoscopic view of the lesion UP: uncinate process, S: septum, MT: middle turbinates

We present a case report of a 65-year-old male patient with type 2 diabetes, hypertension, and ischemic heart disease. The patient presented to the otolaryngology clinic complaining of chronic nasal obstruction that was associated with right eye swelling, diplopia, and progressive decrease in visual acuity of the right eye. Endoscopic examination of the nose was done and revealed a lesion that was smooth on the surface reaching to the middle meatus, covered by mucosa and hard on palpation. The lesion has bounded the ethmoid sinus and the floor of the optical orbit with invasion of the lamina papyracea, extending to the inside of the orbit (Figure 1).

**Figure 2:**
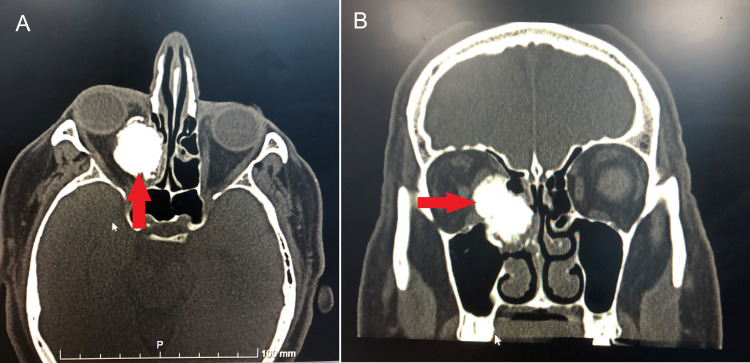
Preoperative computed tomography (CT) scan showing the lesion in cross-sectional view (A) and coronal view (B)

CT scan was done and showed calcified lesion and a glass ground appearance in the right ethmoid air cell eroding the medial wall of the right orbit, displacing the medial rectus muscle and globe laterally and originating from the ethmoid air cell and nasal cavity. In addition, the optic nerve was found to be compressed by the lesion (Figure 2).

**Figure 3:**
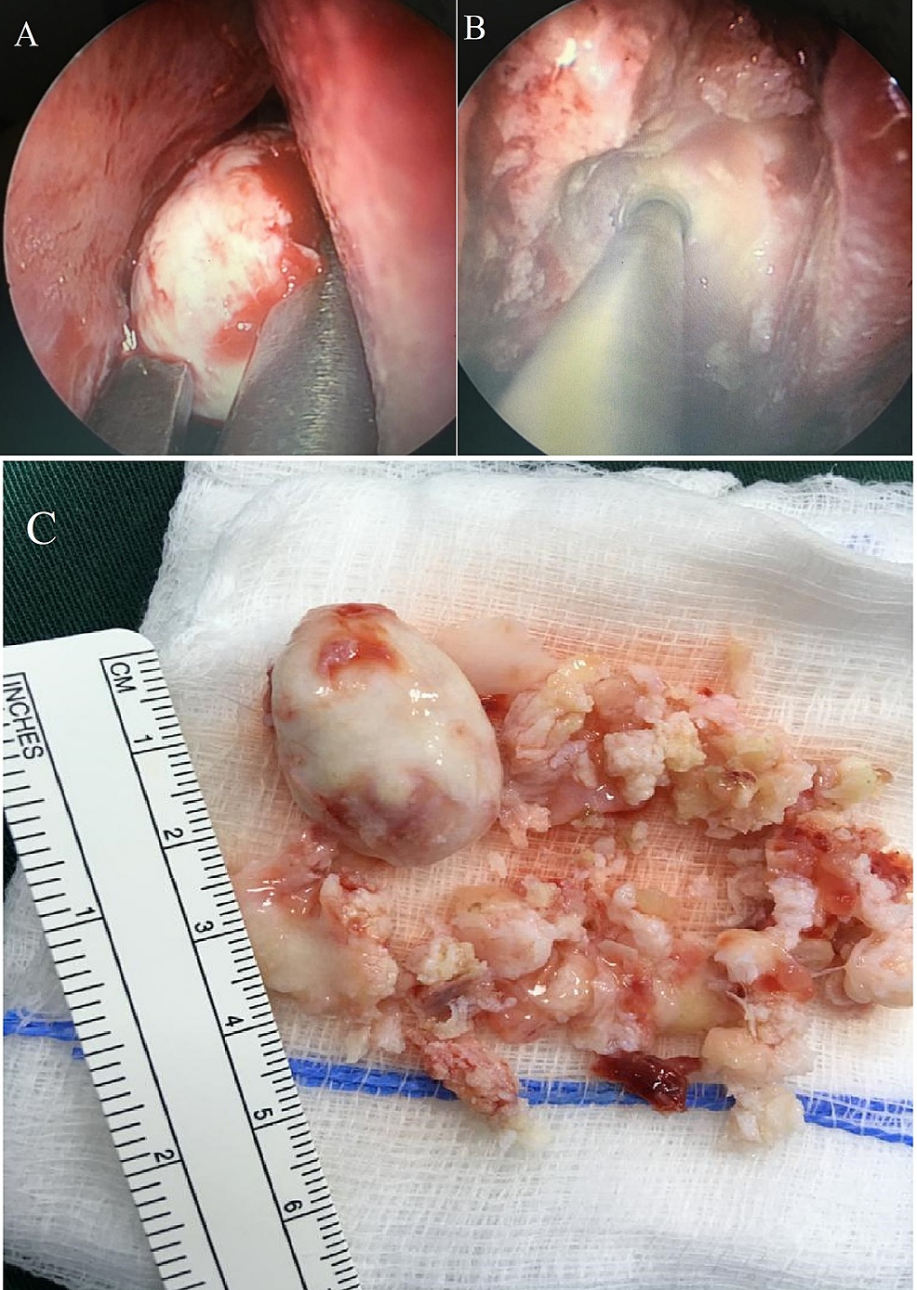
(A) Operative image of the lesion while separating it. (B) Drilling of the lesion. (C) The lesion after excision showing an oval-shaped mass with a length of 3 cm

The lesion was surgically removed using an endoscopic approach with drilling of the nasal part of the mass, and the orbital part was removed as one piece (Figure 3). The patient had an uneventful postoperative hospital course. After excision, the tumor was sent for histopathology in a 10% neutral buffered formalin. Grossly, it was a bony mass of the ethmoid sinus with several pieces of brownish-white bony tissue fragments with a total measurement of 5 x 5 x 1 cm. Furthermore, it included a greyish-white capsulated mass of 3 x 2 x 1.3 cm.

**Figure 4:**
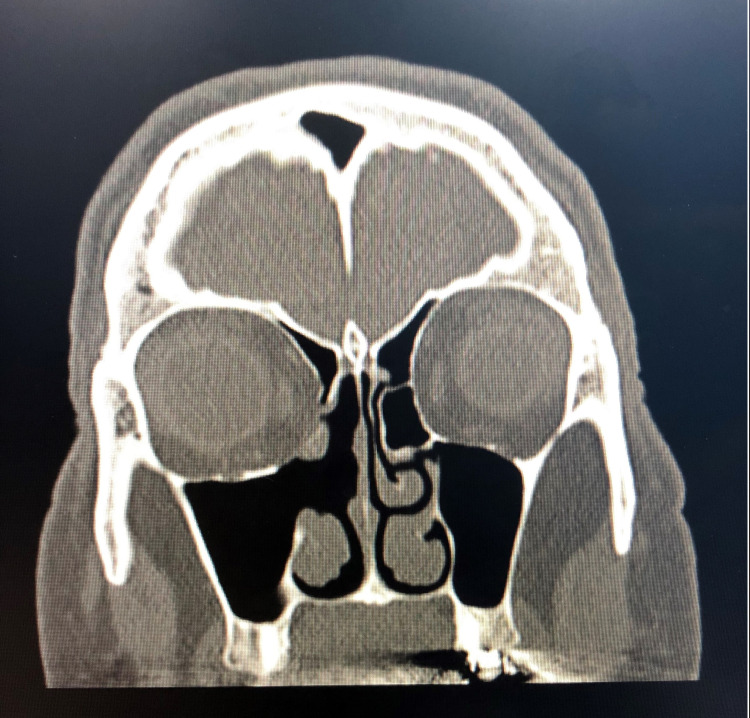
Postoperative coronal CT scan showing complete resolution of the lesion

Microscopically, the histological section showed fragments of sinonasal mucosa, with an underlying well-circumscribed bone-forming lesion composed of a mixture of lamellar and woven bone trabeculae with fibrous stroma. The stroma was surrounding and encasing the bone trabeculae, which were randomly arranged, irregular, and arising directly from the fibrous tissue. The fibrous area was composed of spindle cells in a storiform pattern showing bland, tapered nuclei. Other areas showed nodules of hyaline cartilage with disorganized growth, platelike ossification. A follow-up CT scan was performed after the operation and showed complete resolution with no signs of the lesion (Figure 4).

## Discussion

FD is an uncommon lesions that may occur as an isolated asymptomatic monostotic lesion or a severe polyostotic disease resulting in fractures and deformities [8]. FD of the maxillary and mandible sinuses are the most common sites to be involved, while ethmoidal lesions are rare [9]. FD comprises 2.5% of all bone tumors [10]. It generally affects adolescents and young adults with the commonest presentation of craniofacial malformation and long-lasting headaches [3,10]. Duan et al. performed a retrospective study on a total of 28 patients who were diagnosed with FD [10]. They reported a median age of 31.5 years, while the majority (60%) of the cases were in the young adult group between 20 and 30 years of age. Moreover, the oldest patient that was diagnosed with FD among the group was 69 years old. In addition, 25% reported nasal obstruction as their main symptom, followed by impaired vision (21%), headache (18%), and facial deformity (18%) [10]. Dong et al. studied 77 patients with FD in a retrospective study of paranasal fibro-osseous lesions [3]. Among their group of 77 patients, around 61% were younger than 18 years, with a mean onset of approximately 15 years. Craniofacial deformation was the most commonly reported main symptom (72.7%), followed by headache (16.8%), while only three patients reported nasal obstruction (3.9%) [3]. Compared with our patient, he was 65 years of age with a main presentation of nasal obstruction and long-lasting headache with a progressive decrease in visual acuity. However, the incidence of decreased visual acuity is very rare and can be due to the compression of the optic nerve [3].

Radiologically, CT scans of FD often report a ground glass appearance. However, only a majority of CT scans show this resemblance, making postsurgical histopathology with CT scan the option of choice in the diagnosis of FD [3,10]. Furthermore, a CT scan can also resemble other fibro-osseous lesions, such as osteoma and ossifying fibromas. Dong et al. reported that only 75% of cases that underwent CT scan had a ground glass appearance, while the rest were uncertain diagnoses resembling osteomas and ossifying fibromas [3]. Similarly, our patient CT showed a ground glass appearance of the ethmoid sinus with signs of calcifications.

The main treatment of any paranasal fibroma is total excision of the lesion because the disease itself is usually symptomless until compression of adjacent structures occurs [11]. Endoscopic resection has been reported with very pleasant outcomes, and it is the most common approach taken. The advantages of this approach include direct visualization, a decrease in external deformity, and lower morbidity of more invasive approaches such as the cranial one [11]. On the other hand, radiation therapy has been studied in comparison with a surgical approach. Manes et al. compared radiotherapy to surgical excision. However, they found that it is only a viable choice in the treatment of bony tumors, but not ossifying fibromas [12]. The common outcomes of the direct endoscopic approach include cerebrospinal fluid leakage and recurrence of the lesion. Furthermore, Manes et al. found that the recurrence rate of total excision was around 7% compared with the recurrence rate of 25% in those undergoing subtotal excision, making total excision the procedure of choice in treating such lesions [12]. However, in cases with difficulties in the removal of the whole lesion, the removal of the lesions where pressure is found is warranted [4]. In our patient, complete excision was performed through an endoscopic approach with no recurrence rates even after six months of follow-up.

## Conclusions

Fibrous dysplasia is a fibro-osseous benign lesion of the paranasal sinuses. Its involvement in the ethmoid sinus is rare, but not fatal. Common presentations include headache, eye swelling, and diplopia. Compression of the optic nerve can lead to a progressive decrease in visual acuity unilaterally. Direct endoscopic examination is helpful, and computed tomography is the imaging modality of choice in diagnosing the lesion appearing as a glass ground mass. Total excision of the lesion through a nasal endoscopic approach has the least chances of recurrence with high chances of complete resolution.
